# Four-dimensional generalized difference matrix and some double sequence spaces

**DOI:** 10.1186/s13660-017-1423-y

**Published:** 2017-06-24

**Authors:** Orhan Tuǧ

**Affiliations:** Department of Mathematical Education, Ishik University, Ishik Campus, 100 meter street, Erbil, Iraq

**Keywords:** 46A45, 40C05, four-dimensional generalized difference matrix, matrix domain, double sequence spaces, alpha-dual, beta-dual, gamma-dual, matrix transformations

## Abstract

In this study, I introduce some new double sequence spaces $B(\mathcal {M}_{u})$, $B(\mathcal{C}_{p})$, $B(\mathcal{C}_{bp})$, $B(\mathcal {C}_{r})$ and $B(\mathcal{L}_{q})$ as the domain of four-dimensional generalized difference matrix $B(r,s,t,u)$ in the spaces $\mathcal {M}_{u}$, $\mathcal{C}_{p}$, $\mathcal{C}_{bp}$, $\mathcal{C}_{r}$ and $\mathcal{L}_{q}$, respectively. I show that the double sequence spaces $B(\mathcal{M}_{u})$, $B(\mathcal{C}_{bp})$ and $B(\mathcal{C}_{r})$ are the Banach spaces under some certain conditions. I give some inclusion relations with some topological properties. Moreover, I determine the *α*-dual of the spaces $B(M_{u})$ and $B(\mathcal{C}_{bp})$, the $\beta(\vartheta)$-duals of the spaces $B(M_{u})$, $B(\mathcal{C}_{p})$, $B(\mathcal{C}_{bp})$, $B(\mathcal{C}_{r})$ and $B(\mathcal{L}_{q})$, where $\vartheta\in\{p,bp,r\}$, and the *γ*-dual of the spaces $B(\mathcal{M}_{u})$, $B(\mathcal{C}_{bp})$ and $B(\mathcal{L}_{q})$. Finally, I characterize the classes of four-dimensional matrix mappings defined on the spaces $B(\mathcal{M}_{u})$, $B(\mathcal{C}_{p})$, $B(\mathcal{C}_{bp})$, $B(\mathcal{C}_{r})$ and $B(\mathcal{L}_{q})$ of double sequences.

## Introduction

We denote the set of all complex-valued double sequences by Ω which is a vector space with coordinatewise addition and scalar multiplication. Any subspace of Ω is called a double sequence space. A double sequence $x=(x_{mn})$ of complex numbers is called bounded if $\|x\|_{\infty}=\sup_{m,n\in\mathbb{N}}|x_{mn}|<\infty $, where $\mathbb{N}=\{0,1,2,\ldots\}$. The space of all bounded double sequences is denoted by $\mathcal{M}_{u}$ which is a Banach space with the norm $\|\cdot\|_{\infty}$. Consider the double sequence $x=(x_{mn})\in\Omega$. If for every $\epsilon>0$ there exists a natural number $n_{0}=n_{0}(\epsilon)$ and $l\in\mathbb{C}$ such that $|x_{mn}-l|<\epsilon$ for all $m,n>n_{0}$, then the double sequence *x* is called convergent in Pringsheim’s sense to the limit point *l*, and we write $p\mbox{-} \lim_{m,n\rightarrow\infty}x_{mn}=l$, where $\mathbb {C}$ denotes the complex field. The space of all convergent double sequences in Pringsheim’s sense is denoted by $\mathcal{C}_{p}$. Unlike single sequences, there are such double sequences which are convergent in Pringsheim’s sense but unbounded. That is, the set $\mathcal {C}_{p}-\mathcal{M}_{u}$ is not empty. Actually, following Boos [1], p.16, if we define the sequence $x=(x_{mn})$ by

$$ x_{mn}= \textstyle\begin{cases} n , & m=0, n\in\mathbb{N}; \\ 0 , & m\geq1, n\in\mathbb{N}, \end{cases} $$

then it is obvious that $p\mbox{-} \lim_{m,n\rightarrow\infty}x_{mn}=0$ but $\|x\|_{\infty}=\sup_{m,n\in\mathbb{N}}|x_{mn}|=\infty$, so $x\in \mathcal{C}_{p}-\mathcal{M}_{u} $. Then we can consider the set $\mathcal {C}_{bp}$ of double sequences which are both convergent in Pringsheim’s sense and bounded, i.e., $\mathcal{C}_{bp}=\mathcal{C}_{p}\cap \mathcal{M}_{u}$. Hardy [2] showed that a sequence in the space $\mathcal{C}_{p}$ is said to be regular convergent if it is a single convergent sequence with respect to each index and denoted the space of all such sequences by $\mathcal{C}_{r}$. Moreover, by $\mathcal {C}_{bp0}$ and $\mathcal{C}_{r0}$, we denote the spaces of all double sequences converging to 0 contained in the sequence spaces $\mathcal {C}_{bp}$ and $\mathcal{C}_{r}$, respectively. Móricz [3] proved that $\mathcal{C}_{bp}$, $\mathcal{C}_{bp0}$, $\mathcal {C}_{r}$ and $\mathcal{C}_{r0}$ are Banach spaces with the norm $\| \cdot\|_{\infty}$. By $\mathcal{L}_{q}$, we denote the space of absolutely *q*-summable double sequences corresponding to the space $\ell_{q}$ of *q*-summable single sequences, that is,

$$ \mathcal{L}_{q}:= \biggl\{ x=(x_{kl})\in\Omega:\sum _{k,l}|x_{kl}|^{q}< \infty \biggr\} \quad (1 \leq q< \infty) $$

which is a Banach space with the norm $\|\cdot\|_{q}$ defined by Başar and Sever [4]. Zeltser [5] introduced the space $\mathcal{L}_{u}$ as a special case of the space $\mathcal{L}_{q}$ with $q=1$. Let *λ* be a double sequence space converging with respect to some linear convergence rule $\vartheta\mbox{-} \lim:\lambda\to\mathbb {C}$. The sum of a double series $\sum_{i,j}x_{ij}$ with respect to this rule is defined by $\vartheta\mbox{-} \sum_{i,j}x_{ij}=\vartheta\mbox{-} \lim_{m,n\to\infty}\sum_{i,j=0}^{m,n}x_{ij}$. For short, throughout the text the summations without limits run from 0 to ∞, for instance, $\sum_{i,j}x_{ij}$ means that $\sum_{i,j=0}^{\infty}x_{ij}$.

Here and in what follows, unless stated otherwise, we assume that *ϑ* denotes any of the symbols *p*, *bp* or *r*.

The *α*-dual $\lambda^{\alpha}$, the $\beta(\vartheta)$-dual $\lambda^{\beta(\vartheta)}$ with respect to the *ϑ*-convergence and the *γ*-dual $\lambda^{\gamma}$ of a double sequence space *λ* are respectively defined by

$$\begin{aligned}& \lambda^{\alpha} := \biggl\{ a=(a_{kl})\in\Omega:\sum _{k,l}|a_{kl}x_{kl}|< \infty \text{ for all } x=(x_{kl})\in\lambda \biggr\} , \\& \lambda^{\beta(\vartheta)} := \biggl\{ a=(a_{kl})\in\Omega :\vartheta \mbox{-} \sum_{k,l}a_{kl}x_{kl} \text{ exists for all } x=(x_{kl})\in\lambda \biggr\} , \\& \lambda^{\gamma} := \Biggl\{ a=(a_{kl})\in\Omega:\sup _{m,n\in \mathbb{N}} \Biggl\vert \sum_{k,l=0}^{m,n}a_{kl}x_{kl} \Biggr\vert < \infty \text{ for all } x=(x_{kl})\in\lambda \Biggr\} . \end{aligned}$$

It is easy to see for any two spaces *λ* and *μ* of double sequences that $\mu^{\alpha}\subset\lambda^{\alpha}$ whenever $\lambda\subset\mu$ and $\lambda^{\alpha}\subset\lambda^{\gamma }$. Additionally, it is known that the inclusion $\lambda^{\alpha }\subset\lambda^{\beta(\vartheta)}$ holds, while the inclusion $\lambda^{\beta(\vartheta)}\subset\lambda^{\gamma}$ does not hold since the *ϑ*-convergence of the double sequence of partial sums of a double series does not imply its boundedness.

Let *λ* and *μ* be two double sequence spaces and $A=(a_{mnkl})$ be any four-dimensional complex infinite matrix. Then we say that *A* defines a matrix mapping from *λ* into *μ*, and we write $A:\lambda\to\mu$ if for every sequence $x=(x_{kl})\in \lambda$ the *A*-transform $Ax=\{(Ax)_{mn}\}_{m,n\in\mathbb{N}}$ of *x* exists and it is in *μ* where

1.1 $$ (Ax)_{mn}=\vartheta\mbox{-} \sum _{k,l}a_{mnkl}x_{kl}\quad \text{for each } m,n\in\mathbb{N}. $$

We define *ϑ*-summability domain $\lambda_{A}^{(\vartheta)}$ of *A* in a space *λ* of double sequences by

$$ \lambda_{A}^{(\vartheta)}= \biggl\{ x=(x_{kl})\in\Omega: Ax= \biggl(\vartheta\mbox{-} \sum_{k,l}a_{mnkl}x_{kl} \biggr)_{m,n\in\mathbb {N}}\text{ exists and is in }\lambda \biggr\} . $$

We say with notation (1.1) that *A* maps the space *λ* into the space *μ* if $\lambda\subset\mu_{A}^{(\vartheta)}$, and we denote the set of all four-dimensional matrices, transforming the space *λ* into the space *μ*, by $(\lambda:\mu)$. Thus, $A=(a_{mnkl})\in(\lambda:\mu)$ if and only if the double series on the right-hand side of (1.1) converges in the sense of *ϑ* for each $m,n\in\mathbb{N}$, i.e., $A_{mn}\in\lambda ^{\beta(\vartheta)}$ for all $m,n\in\mathbb{N}$ and every $x\in \lambda$, and we have $Ax\in\mu$ for all $x\in\lambda$, where $A_{mn}=(a_{mnkl})_{k,l\in\mathbb{N}}$ for all $m,n\in\mathbb{N}$. We say that a four-dimensional matrix *A* is $\mathcal{C}_{\vartheta }$-conservative if $\mathcal{C}_{\vartheta}\subset(\mathcal {C}_{\vartheta})_{A}$, and is $\mathcal{C}_{\vartheta}$-regular if it is $\mathcal{C}_{\vartheta}$-conservative and

$$ \vartheta\mbox{-} \lim Ax=\vartheta\mbox{-} \lim _{m,n\to\infty}(Ax)_{mn}=\vartheta \mbox{-} \lim _{m,n\to\infty}x_{mn},\quad \text{where } x=(x_{mn})\in\mathcal {C}_{\vartheta}. $$

Adams [6] defined that the four-dimensional infinite matrix $A=(a_{mnkl})$ is called a triangular matrix if $a_{mnkl}=0$ for $k>m$ or $l>n$ or both. We also say by [6] that a triangular matrix $A=(a_{mnkl})$ is said to be a triangle if $a_{mnmn}\neq0$ for all $m,n\in\mathbb{N}$. Moreover, by referring to Cooke [7], Remark (a), p.22, we can say that every triangle matrix has a unique inverse which is also a triangle.

Let $r,s,t,u\in\mathbb{R}\setminus\{0\}$. Then the four-dimensional generalized difference matrix $B(r,s, t,u)=\{b_{mnkl}(r,s,t,u)\}$ is defined by

$$ b_{mnkl}(r,s,t,u):= \textstyle\begin{cases} su, & (k,l)=(m-1,n-1), \\ st, & (k,l)=(m-1,n), \\ ru, & (k,l)=(m,n-1), \\ rt, & (k,l)=(m,n) \\ 0, &\text{otherwise} \end{cases} $$

for all $m,n,k,l\in\mathbb{N}$. Therefore, the $B(r,s,t,u)$-transform of a double sequence $x=(x_{mn})$ is given by

1.2 $$\begin{aligned} y_{mn} :=&\bigl\{ B(r,s,t,u)x\bigr\} _{mn}=\sum _{k,l}b_{mnkl}(r,s,t,u)x_{kl} \\ =&sux_{m-1,n-1}+stx_{m-1,n}+rux_{m,n-1}+rtx_{mn} \end{aligned}$$

for all $m,n\in\mathbb{N}$. Thus, we have the inverse $B^{-1}(r,s,t,u)=F(r,s,t,u)=\{f_{mnkl}(r,s,t,u)\}$ as follows:

$$ f_{mnkl}(r,s,t,u):= \textstyle\begin{cases} \frac{(-s/r)^{m-k}(-u/t)^{n-l}}{rt}, & 0\leq k\leq m, 0\leq l\leq n, \\ 0, & \text{otherwise} \end{cases} $$

for all $m,n,k,l\in\mathbb{N}$. Therefore, we can obtain $x=(x_{mn})$ by applying the inverse matrix $F(r,s,t,u)$ to (1.2) that

1.3 $$ x_{mn}=\frac{1}{rt}\sum _{k,l=0}^{m,n} \biggl(\frac{-s}{r} \biggr)^{m-k} \biggl(\frac{-u}{t} \biggr)^{n-l}y_{kl} \quad \text{for all } m,n\in\mathbb{N}. $$

Throughout the paper, we suppose that the terms of double sequence $x=(x_{mn})$ and $y=(y_{mn})$ are connected with relation (1.2). If $p\mbox{-} \lim\{B(r,s,t,u)x\}_{mn}=l$, then the sequence $x=(x_{mn})$ is said to be $B(r,s,t,u)$ convergent to *l*. Note that in the case $r=t=1$ and $s=u=-1$ for all $m,n\in\mathbb{N}$, the four-dimensional generalized difference matrix $B(r,s,t,u)$ is reduced to the four-dimensional difference matrix $\Delta=B(1, -1, 1, -1)$.

## Some new spaces of double sequences

In this section, we define the double sequence spaces $B(\mathcal {M}_{u})$, $B(\mathcal{C}_{p})$, $B(\mathcal{C}_{bp})$, $B(\mathcal {C}_{r})$ and $B(\mathcal{L}_{q})$ as the domain of four-dimensional generalized difference matrix $B(r,s,t,u)$ in the double sequence spaces $\mathcal{M}_{u}$, $\mathcal{C}_{p}$, $\mathcal{C}_{bp}$, $\mathcal{C}_{r}$ and $\mathcal{L}_{q}$, respectively, that is,

$$\begin{aligned}& B(\mathcal{M}_{u}) := \Bigl\{ x=(x_{mn})\in\Omega: \sup _{m,n\in \mathbb{N}} \bigl\vert \bigl\{ B(r,s,t,u)x\bigr\} _{mn} \bigr\vert < \infty \Bigr\} , \\& B(\mathcal{C}_{p}) := \Bigl\{ x=(x_{mn})\in\Omega:\exists l \in\mathbb {C} \ni p\mbox{-} \lim_{m,n\to\infty} \bigl\vert \bigl\{ B(r,s,t,u)x\bigr\} _{mn}-l \bigr\vert =0 \Bigr\} , \\& B(\mathcal{C}_{bp}) := \bigl\{ x=(x_{mn})\in \Omega:B(r,s,t,u)x\in \mathcal{C}_{bp} \bigr\} , \\& B(\mathcal{C}_{r}) := \bigl\{ x=(x_{mn})\in \Omega:B(r,s,t,u)x\in \mathcal{C}_{r} \bigr\} , \\& B(\mathcal{L}_{q}) := \biggl\{ x=(x_{mn})\in\Omega: \sum _{m,n} \bigl\vert \bigl\{ B(r,s,t,u)x\bigr\} _{mn} \bigr\vert ^{q}< \infty \biggr\} , \quad 0< q< \infty. \end{aligned}$$

Then we give some topological properties and inclusion relations.

### Theorem 2.1

*The double sequence spaces* $B(\mathcal{M}_{u})$, $B(\mathcal{C}_{bp})$ *and* $B(\mathcal{C}_{r})$ *are linear Banach spaces with coordinatwise addition and scalar multiplication*, *and are linearly norm isomorphic to the spaces* $\mathcal{M}_{u}$, $\mathcal{C}_{bp}$ *and* $\mathcal{C}_{r}$, *respectively*, *with the norm*

2.1 $$ \|x\|_{B(\mathcal{M}_{u})}=\sup_{k,l\in\mathbb {N}}|sux_{k-1,l-1}+stx_{k-1,l}+rux_{k,l-1}+rtx_{kl}|. $$

### Proof

We only prove the theorem for the space $B(\mathcal{M}_{u})$ since it can be shown in the same way for the other spaces. It is easy to show the linearity of the space, so we omit the details. Let us consider a Cauchy sequence $x^{j}=\{x_{mn}^{q}\}_{m,n\in\mathbb{N}}\in B(\mathcal {M}_{u})$ in order to show that the space $B(\mathcal{M}_{u})$ is a Banach space with the norm $\|x\|_{B(\mathcal{M}_{u})}$ defined by (2.1). Then, for a given $\epsilon>0$, there exists a positive integer $N(\epsilon)\in\mathbb{N}$ such that

2.2 $$\begin{aligned} \bigl\Vert x^{j}-x^{i} \bigr\Vert _{B(\mathcal{M}_{u})} =&\sup_{m,n\in\mathbb{N}} \bigl\vert \bigl\{ B(r,s,t,u)x^{j}\bigr\} _{mn}-\bigl\{ B(r,s,t,u)x^{i} \bigr\} _{mn} \bigr\vert \\ < &\epsilon\quad \text{for all } i,j>N( \epsilon). \end{aligned}$$

Then we have power to say that $\{(B(r,s,t,u)x^{j})_{mn}\}_{j\in \mathbb{N}}$ is a Cauchy sequence in $\mathcal{M}_{u}$ for each $m,n\in \mathbb{N}$. Since $\mathcal{M}_{u}$ is complete, it converges, say

$$ \bigl\{ B(r,s,t,u)x^{j}\bigr\} _{mn}\to\bigl\{ B(r,s,t,u)x \bigr\} _{mn} \quad \text{as }p\to \infty. $$

By taking limit as $p\to\infty$ on equality (2.2), we have that

$$ \bigl\vert \bigl\{ B(r,s,t,u)x^{j}\bigr\} _{mn}-\bigl\{ B(r,s,t,u)x\bigr\} _{mn} \bigr\vert < \epsilon\quad \text{for all } m,n\in\mathbb{N}. $$

Moreover, since $\{\{B(r,s,t,u)x^{j}\}_{mn}\}\in\mathcal{M}_{u}$, there exists a positive real number *K* such that

$$ \sup_{m,n\in\mathbb{N}} \bigl\vert \bigl\{ B(r,s,t,u)x^{j}\bigr\} _{mn} \bigr\vert \leq K. $$

Hence, the following inequality

$$\begin{aligned} \bigl\vert \bigl\{ B(r,s,t,u)x\bigr\} _{mn} \bigr\vert \leq& \bigl\vert \bigl\{ B(r,s,t,u)x^{j}\bigr\} _{mn}-\bigl\{ B(r,s,t,u)x \bigr\} _{mn} \bigr\vert + \bigl\vert \bigl\{ B(r,s,t,u)x^{j} \bigr\} _{mn} \bigr\vert \\ < &\epsilon+K \end{aligned}$$

is satisfied. Therefore, by taking supremum over $m,n\in\mathbb{N}$ for all the results obtained above gives that $B(r,s,t,u)x\in\mathcal {M}_{u}$, that is, $x\in B(\mathcal{M}_{u})$. We read from here that the space $B(\mathcal{M}_{u})$ is a linear Banach space with the norm $\| \cdot\|_{B(\mathcal{M}_{u})}$ defined by (2.1). Since the proof can be given in the same way for the other spaces, we only show here that $B(\mathcal{M}_{u})$ is linearly isomorphic to the space $\mathcal{M}_{u}$. With the notation of (1.2), define the transformation *T* from $B(\mathcal{M}_{u})$ to $\mathcal{M}_{u}$ by $x\mapsto Tx=y=B(r,s,t,u)x$. Then it is trivial that *T* is linear and injective. Let $y=(y_{kl})\in\mathcal{M}_{u}$ and define $x=(x_{mn})$ via the sequence *y* by relation (1.3) for all $m,n\in\mathbb {N}$. Therefore, we see by (1.2) that

$$\begin{aligned} \bigl\{ B(r,s,t,u)x\bigr\} _{mn} =&sux_{m-1,n-1}+stx_{m-1,n}+rux_{m,n-1}+rtx_{mn} \\ =&su\sum_{k,l=0}^{m-1,n-1} \biggl( \frac{-s}{r} \biggr)^{m-k-1} \biggl(\frac{-u}{t} \biggr)^{n-l-1}\frac{y_{kl}}{rt} \\ &{}+st\sum_{k,l=0}^{m-1,n} \biggl(\frac{-s}{r} \biggr)^{m-k-1} \biggl(\frac {-u}{t} \biggr)^{n-l}\frac{y_{kl}}{rt} \\ &{}+ru\sum_{k,l=0}^{m,n-1} \biggl( \frac{-s}{r} \biggr)^{m-k} \biggl(\frac{-u}{t} \biggr)^{n-l-1}\frac{y_{kl}}{rt} \\ &{}+rt\sum_{k,l=0}^{m,n} \biggl(\frac{-s}{r} \biggr)^{m-k} \biggl(\frac {-u}{t} \biggr)^{n-l}\frac{y_{kl}}{rt} \\ =&y_{mn} \end{aligned}$$

for all $m,n\in\mathbb{N}$, which leads us to the consequence that

$$ \|x\|_{B(\mathcal{M}_{u})}=\sup_{m,n\in\mathbb{N}} \bigl\vert \bigl\{ B(r,s,t,u)x\bigr\} _{mn} \bigr\vert =\sup_{m,n\in\mathbb{N}}|y_{mn}|= \| y\|_{\infty}< \infty. $$

This means that $x=(x_{mn})$ defined by (1.3) is in the space $B(\mathcal{M}_{u})$, i.e., *T* is surjective and is norm-preserving.

This concludes the proof of the theorem. □

### Theorem 2.2

*The inclusion* $\mathcal{M}_{u}\subset B(\mathcal{M}_{u})$ *strictly holds*.

### Proof

Firstly, we show that the inclusion $\mathcal{M}_{u}\subset B(\mathcal {M}_{u})$ holds. For this, when we take a double sequence $x=(x_{mn})\in \mathcal{M}_{u}$, then there exists a positive real number *K* such that $\sup_{m,n\in\mathbb{N}}|x_{mn}|\leq K$. Therefore, one can easily see that

$$\begin{aligned} \begin{aligned} \sup_{m,n\in\mathbb{N}} \bigl\vert \bigl\{ B(r,s,t,u)x\bigr\} _{mn} \bigr\vert &=\sup_{m,n\in \mathbb{N}} \vert sux_{m-1,n-1}+stx_{m-1,n}+rux_{m,n-1}+rtx_{mn} \vert \\ &\leq\bigl( \vert su \vert +|st|+|ru|+|rt|\bigr)K< \infty. \end{aligned} \end{aligned}$$

This means that the double sequence $x=(x_{mn})\in B(\mathcal{M}_{u})$, that is, the inclusion $\mathcal{M}_{u}\subset B(\mathcal{M}_{u})$ holds.

Now, we prove that this inclusion is strict. That is, the set $B(\mathcal{M}_{u})\setminus\mathcal{M}_{u}$ is not empty. Let us consider the double sequence $x=(x_{mn})$ defined by $x_{mn}=(-1)^{m+n}(m+1)(n+1)$ for all $m,n\in\mathbb{N}$. It is obvious that *x* is not in $\mathcal{M}_{u}$. If we take $r=t=s=u$, then we obtain $\{B(r,s,t,u)\}$-transform of *x* as

$$\begin{aligned} \bigl\{ B(r,r,r,r)x\bigr\} _{mn} =&r^{2}\bigl[(-1)^{m+n-2}mn+(-1)^{m+n-1}m(n+1) \\ &{}+(-1)^{m+n-1}(m+1)n+(-1)^{m+n}(m+1) (n+1)\bigr] \\ =&(-1)^{m+n}r^{2} \end{aligned}$$

which gives the fact that $B(r,r,r,r)x\in\mathcal{M}_{u}$. This completes the proof. □

### Theorem 2.3

*The inclusion* $\mathcal{C}_{p}\subset B(\mathcal{C}_{p})$ *strictly holds*.

### Proof

For the first step of the proof, we show that the inclusion $\mathcal {C}_{p}\subset B(\mathcal{C}_{p})$ holds. Let us take a sequence $x=(x_{mn})\in\mathcal{C}_{p}$. Then there exists a complex number *l* such that $p\mbox{-} \lim_{m,n\to\infty}|x_{mn}-l|=0$. Then we have by taking limit of the $B(r,s,t,u)$-transform of *x* as $m,n\to\infty$ in Pringsheim’s sense

$$\begin{aligned} p\mbox{-} \lim_{m,n\to\infty}\bigl\{ B(r,s,t,u)x\bigr\} _{mn} =&p\mbox{-} \lim_{m,n\to\infty } (sux_{m-1,n-1}+stx_{m-1,n}+rux_{m,n-1}+rtx_{mn} ) \\ =&su \Bigl(p\mbox{-} \lim_{m,n\to\infty}x_{m-1,n-1} \Bigr)+st \Bigl(p\mbox{-} \lim_{m,n\to\infty}x_{m-1,n} \Bigr) \\ &{}+ru \Bigl(p\mbox{-} \lim_{m,n\to\infty}x_{m,n-1} \Bigr)+rt \Bigl(p\mbox{-} \lim_{m,n\to\infty}x_{mn} \Bigr). \end{aligned}$$

Since $x\in\mathcal{C}_{p}$, then all the subsequences of *x* are also convergent. Thus, $B(r,s,t,u)x\in C_{p}$, i.e., $x\in B(\mathcal{C}_{p})$.

To prove the fact that the inclusion $\mathcal{C}_{p}\subset B(\mathcal{C}_{p})$ is strict, we should show that the set $B(\mathcal {C}_{p})\setminus\mathcal{C}_{p}$ is not empty. Let us consider the double sequence $x=(x_{mn})$ defined by $x_{mn}=(mn)/(rt)$ for all $m,n\in\mathbb{N}$. If we take $s=-r$, $u=-t$, then we have

$$\begin{aligned} \bigl\{ B(r,-r,t,-t)x\bigr\} _{mn} =&rtx_{m-1,n-1}-rtx_{m-1,n}-rtx_{m,n-1}+rtx_{mn} \\ =&rt\frac{(m-1)(n-1)}{rt}-rt\frac{(m-1)n}{rt}-rt\frac {m(n-1)}{rt}+rt \frac{mn}{rt} \\ =&1 \end{aligned}$$

for all $m,n\in\mathbb{N}$. Thus, one can easily observe that $x=(x_{mn})\notin\mathcal{C}_{p}$. But, $p\mbox{-} \lim_{m,n\to\infty}\{ B(r,s, t,u)x\}_{mn}=1$, that is, $x\in B(\mathcal{C}_{p})$. This step completes the proof. □

### Theorem 2.4

*The inclusion* $\mathcal{C}_{bp}\subset B(\mathcal{C}_{bp})$ *strictly holds*.

### Proof

This is a natural consequence of Theorems 2.2 and 2.3. So, we omit the details. □

### Theorem 2.5

*The inclusion* $\mathcal{L}_{q}\subset B(\mathcal{L}_{q})$ *strictly holds*, *where* $1\leq q<\infty$.

### Proof

Let us take a double sequence $x=(x_{mn})\in\mathcal{L}_{q}$ with $1\leq q<\infty$. Then $\sum_{m,n}|x_{mn}|^{q}<\infty$. Now, we have

$$\begin{aligned} \biggl[\sum_{m,n} \bigl\vert \bigl\{ B(r,s,t,u)x \bigr\} _{mn} \bigr\vert ^{q} \biggr]^{1/q} =& \biggl(\sum_{m,n} \vert sux_{m-1,n-1}+stx_{m-1,n}+rux_{m,n-1}+rtx_{mn} \vert ^{q} \biggr)^{1/q} \\ \leq&|su| \biggl(\sum_{m,n} \vert x_{m-1,n-1} \vert ^{q} \biggr)^{1/q}+|st| \biggl(\sum _{m,n} \vert x_{m-1,n} \vert ^{q} \biggr)^{1/q} \\ &{}+|ru| \biggl(\sum_{m,n} \vert x_{m,n-1} \vert ^{q} \biggr)^{1/q}+|rt| \biggl(\sum _{m,n} \vert x_{mn} \vert ^{q} \biggr)^{1/q}< \infty, \end{aligned}$$

which says that $B(r,s,t,u)x\in\mathcal{L}_{q}$, i.e., $x\in B(\mathcal{L}_{q})$.

In order to prove the fact that the inclusion is strict, we should define a double sequence belonging to $B(\mathcal{L}_{q})$ but not to $\mathcal{L}_{q}$. Let us define the double sequence $x=(x_{mn})$ by

$$ x_{mn}= \biggl(\frac{-s}{r} \biggr)^{m} \biggl( \frac{-u}{t} \biggr)^{n}\frac{1}{rt} $$

for all $m,n\in\mathbb{N}$. If $(\frac{-s}{r} )>1$ or $(\frac{-u}{t} )>1$, or both, then it is obvious that $x\notin\mathcal{L}_{q}$. But, under the same restrictions, we have

$$\begin{aligned} \sum_{m,n}\bigl|\bigl\{ B(r,s,t,u)x\bigr\} _{mn}\bigr|^{q} =&\sum_{m,n} \biggl\vert su \biggl(\frac {-s}{r} \biggr)^{m-1} \biggl( \frac{-u}{t} \biggr)^{n-1}\frac {1}{rt}+st \biggl( \frac{-s}{r} \biggr)^{m-1} \biggl(\frac{-u}{t} \biggr)^{n}\frac{1}{rt} \\ &{}+ru \biggl(\frac{-s}{r} \biggr)^{m} \biggl(\frac{-u}{t} \biggr)^{n-1}\frac{1}{rt}+rt \biggl(\frac{-s}{r} \biggr)^{m} \biggl(\frac {-u}{t} \biggr)^{n} \frac{1}{rt} \biggr\vert ^{q}=0. \end{aligned}$$

This says that $B(r,s,t,u)x\in\mathcal{L}_{q}$, i.e., $x\in B(\mathcal {L}_{q})$. This completes the proof. □

### Theorem 2.6

*Let* $1\leq q< q_{1}<\infty$. *Then the inclusion* $B(\mathcal{L}_{q})\subset B(\mathcal{L}_{q_{1}})$ *holds*.

### Proof

Let us take a double sequence $x=(x_{mn})\in B(\mathcal{L}_{q})$ which implies that $Bx\in\mathcal{L}_{q}$. Since the inclusion $\mathcal {L}_{q}\subset\mathcal{L}_{q_{1}}$ holds for $1\leq q< q_{1}<\infty$, by Başar and Sever [4], we have the fact that $Bx\in \mathcal{L}_{q_{1}}$. Hence, $x\in B(\mathcal{L}_{q_{1}})$, as desired. □

### Theorem 2.7

*The set* $B(\mathcal{C}_{p})$ *becomes a linear space with coordinatewise additions and scalar multiplication which is linearly isomorphic to the space* $\mathcal{C}_{p}$, *and* $B(\mathcal{C}_{p})$ *is a complete seminormed space with the seminorm*

$$ \|x\|_{B(\mathcal{C}_{p})}=\lim_{k\to\infty} \Bigl(\sup _{m,n\geq k} \bigl\vert \bigl\{ B(r,s,t,u)x\bigr\} _{mn} \bigr\vert \Bigr). $$

### Proof

The proof of the theorem is similar to the proof of Theorem 2.1. So, we omit the details. □

### Theorem 2.8

*The set* $B(\mathcal{L}_{q})$ *is a linear space with coordinatewise addition and scaler multiplication*, *and the following statements hold*.

(i) *If* $0< q<1$, *then* $B(\mathcal{L}_{q})$ *is a complete* *q*-*normed space with the norm*
  $$ \|x\hat{\|}_{B(\mathcal{L}_{q})}=\sum_{m,n} \bigl\vert \bigl\{ B(r,s,t,u)x\bigr\} _{mn} \bigr\vert ^{q} $$
  *which is* *q*-*norm isomorphic to the space* $\mathcal{L}_{q}$.
(ii) *If* $1\leq q<\infty$, *then* $B(\mathcal{L}_{q})$ *is a Banach space with the norm*
  $$ \|x\|_{B(\mathcal{L}_{q})}= \biggl[\sum_{m,n} \bigl\vert \bigl\{ B(r,s,t,u)x\bigr\} _{mn} \bigr\vert ^{q} \biggr]^{1/q} $$
  *which is norm isomorphic to the space* $\mathcal{L}_{q}$.

### Proof

(i) To show the linearity of the space $B(\mathcal{L}_{q})$ which is a *q*-normed space with the given norm is a routine verification. So, we omit the details. Let us take a Cauchy sequence $x^{i}= \{ x_{mn}^{(i)} \}_{m,n\in\mathbb{N}}$ for every fixed $i\in \mathbb{N}$ in the space $B(\mathcal{L}_{q})$. Then, for a given $\epsilon>0$, there exists a positive real number $N(\epsilon)>0$ such that

$$ \bigl\Vert x^{i}-x^{j}\hat{ \bigr\Vert }_{B(\mathcal{L}_{q})}=\sum_{m,n} \bigl\vert \bigl\{ B(r,s,t,u)x^{i}\bigr\} _{mn}-\bigl\{ B(r,s,t,u)x^{j} \bigr\} _{mn} \bigr\vert ^{q}< \epsilon $$

is satisfied for all $i,j\geq N(\epsilon)$. Then we conclude that $\{\{B(r,s,t,u)x^{i}\}_{mn} \}_{i\in\mathbb{N}}$ is a Cauchy sequence for each fixed $m,n\in\mathbb{N}$. It is known by Part (i) of Theorem 2.1 of Yeşilkayagil and Başar [8] that the space $\mathcal{L}_{q}$ is a complete *q*-normed space. Then the Cauchy sequence $\{(Bx^{i})_{mn} \}_{i\in\mathbb{N}}$ is convergent in the space $\mathcal{L}_{q}$, as $i\to\infty$, that is, there exists a sequence $B(r,s,t,u)x\in\mathcal{L}_{q}$ such that

$$ \bigl\vert \bigl\{ B(r,s,t,u)x^{i}\bigr\} _{mn}-\bigl\{ B(r,s,t,u)x\bigr\} _{mn} \bigr\vert < \epsilon $$

for all $m,n\in\mathbb{N}$. Furthermore, since the $\{\{ B(r,s,t,u)x^{i}\}_{mn} \}\in\mathcal{L}_{q}$ for each fixed $i\in \mathbb{N}$, there exists a positive real number $M>0$ such that $\sum_{m,n}|\{B(r,s,t,u)x^{i}\}_{mn}|^{q}\leq M$. Therefore, we have

$$\begin{aligned} \sum_{m,n} \bigl\vert (Bx)_{mn} \bigr\vert ^{q} \leq&\sum_{m,n} \bigl( \bigl\vert \bigl\{ B(r,s,t,u)x^{i}\bigr\} _{mn}-\bigl\{ B(r,s,t,u)x \bigr\} _{mn} \bigr\vert + \bigl\vert \bigl\{ B(r,s,t,u)x^{i} \bigr\} _{mn} \bigr\vert \bigr)^{q} \\ \leq&\sum_{m,n} \bigl\vert \bigl\{ B(r,s,t,u)x^{i}\bigr\} _{mn}-\bigl\{ B(r,s,t,u)x\bigr\} _{mn} \bigr\vert ^{q}+\sum_{m,n} \bigl\vert \bigl\{ B(r,s,t,u)x^{i}\bigr\} _{mn} \bigr\vert ^{q} \\ < &\epsilon+M, \end{aligned}$$

which means that $B(r,s,t,u)x\in\mathcal{L}_{q}$, that is, $x\in B(\mathcal{L}_{q})$. The last conclusion says that the space $B(\mathcal {L}_{q})$ is a complete *q*-normed space.

Now, we should define a transform from $B(\mathcal{L}_{q})$ to $\mathcal {L}_{q}$ which is a norm-preserving bijection. Let us consider the transformation *T* used in the proof of the second part of Theorem 2.1 with $B(\mathcal{L}_{q})$ and $\mathcal{L}_{q}$ instead of $B(\mathcal{M}_{u})$ and $\mathcal{M}_{u}$, respectively. It is easy to see that *T* is linear and bijective. Let $y=(y_{mn})\in\mathcal {L}_{q}$ and define $x=(x_{mn})$ by relation (1.3). Then we derive by taking summation over $m,n\in\mathbb{N}$ on the following inequality:

$$\begin{aligned} \bigl\vert \bigl\{ B(r,s,t,u)x\bigr\} _{mn} \bigr\vert ^{q} =& \vert sux_{m-1,n-1}+stx_{m-1,n}+rux_{m,n-1}+rtx_{mn} \vert ^{q} \\ =&\biggl|\frac{su}{rt}\sum_{k,l=0}^{m-1,n-1} \biggl(\frac {-s}{r} \biggr)^{m-k-1} \biggl(\frac{-u}{t} \biggr)^{n-l-1}y_{kl} \\ &{}+\frac {st}{rt}\sum _{k,l=0}^{m-1,n} \biggl(\frac{-s}{r} \biggr)^{m-k-1} \biggl(\frac{-u}{t} \biggr)^{n-l}y_{kl} \\ &{}+\frac{ru}{rt}\sum_{k,l=0}^{m,n-1} \biggl(\frac{-s}{r} \biggr)^{m-k} \biggl(\frac{-u}{t} \biggr)^{n-l}y_{kl} \\ &{}+\frac{rt}{rt}\sum _{k,l=0}^{m,n} \biggl(\frac{-s}{r} \biggr)^{m-k} \biggl(\frac {-u}{t} \biggr)^{n-l}y_{kl}\biggr|^{q} \\ =&|y_{mn}|^{q} \end{aligned}$$

that $\|B(r,s,t,u)x\hat{\|}_{B(\mathcal{L}_{q})}=\|y\hat{\| }_{q}$, that is, $x\in B(\mathcal{L}_{q})$. Thus, *T* is surjective. This concludes the proof of Part (i).

Since Part (ii) can be proved in a similar way, we omit the details. □

## The alpha-, beta- and gamma-duals of the new double sequence spaces

In this present section, we calculate the *α*-dual of the spaces $B(\mathcal{M}_{u})$ and $B(\mathcal{C}_{bp})$, the $\beta(\vartheta )$-duals of the spaces $B(\mathcal{M}_{u})$, $B(\mathcal{C}_{p})$, $B(\mathcal{C}_{bp})$, $B(\mathcal{C}_{r})$ and $B(\mathcal{L}_{q})$ and the *γ*-dual of the spaces $B(\mathcal{M}_{u})$, $B(\mathcal {C}_{bp})$ and $B(\mathcal{L}_{q})$.

### Theorem 3.1

*The* *α*-*dual of the spaces* $B(\mathcal{M}_{u})$ *and* $B(\mathcal {C}_{bp})$ *is the space* $\mathcal{L}_{u}$.

### Proof

To prove the equality $\{B(\mathcal{M}_{u}) \}^{\alpha }=\mathcal{L}_{u}$, we should show that the inclusions $\mathcal {L}_{u}\subset \{B(\mathcal{M}_{u}) \}^{\alpha}$ and $\{ B(\mathcal{M}_{u}) \}^{\alpha}\subset\mathcal{L}_{u}$ hold. Let us take a sequence $a=(a_{mn})\in\mathcal{L}_{u}$ and $x=(x_{mn})\in B(\mathcal{M}_{u})$. Then there exists a double sequence $y=(y_{mn})\in \mathcal{M}_{u}$ with relation (1.2) that there exists a positive real number $M>0$ such that $\sup_{m,n\in\mathbb {N}}|y_{mn}|\leq M$. If $\vert s/r \vert , \vert u/t \vert <1$, then we have the following inequality:

$$\begin{aligned} \sum_{m,n}|a_{mn}x_{mn}| =&\sum _{m,n} \vert a_{mn} \vert \Biggl\vert \sum_{k,l=0}^{m,n} \biggl(\frac{-s}{r} \biggr)^{m-k} \biggl(\frac {-u}{t} \biggr)^{n-l} \frac{y_{kl}}{rt} \Biggr\vert \\ \leq&\frac{1}{|rt|}\sum_{m,n} \vert a_{mn} \vert \sum_{k,l=0}^{m,n} \biggl\vert \biggl(\frac{-s}{r} \biggr)^{m-k} \biggl( \frac {-u}{t} \biggr)^{n-l} \biggr\vert |y_{kl}| \\ \leq&\frac{M}{|rt|}\sum_{m,n} \vert a_{mn} \vert \sum_{k,l=0}^{m,n} \biggl\vert \frac{-s}{r} \biggr\vert ^{m-k} \biggl\vert \frac {-u}{t} \biggr\vert ^{n-l} \\ =&\frac{M}{|rt|}\sum_{m,n} \vert a_{mn} \vert \biggl(\frac{1- \vert \frac{s}{r} \vert ^{m-k}}{1- \vert \frac{s}{r} \vert } \biggr) \biggl( \frac{1- \vert \frac{u}{t} \vert ^{n-l}}{1- \vert \frac {u}{t} \vert } \biggr) \\ =&\frac{M}{|rt|} \biggl(\frac{1}{1- \vert \frac{s}{r} \vert } \biggr) \biggl( \frac{1}{1- \vert \frac{u}{t} \vert } \biggr)\sum_{m,n} \vert a_{mn} \vert \biggl(1- \biggl\vert \frac{s}{r} \biggr\vert ^{m+1} \biggr) \biggl(1- \biggl\vert \frac{u}{t} \biggr\vert ^{n+1} \biggr) \\ =&\frac{M}{|rt|} \biggl(\frac{1}{1- \vert \frac{s}{r} \vert } \biggr) \biggl( \frac{1}{1- \vert \frac{u}{t} \vert } \biggr)\sum_{m,n} \vert a_{mn} \vert \\ < &\infty \end{aligned}$$

which says that $a=(a_{mn})\in \{B(\mathcal{M}_{u}) \} ^{\alpha}$. Hence, the inclusion $\mathcal{L}_{u}\subset \{ B(\mathcal{M}_{u}) \}^{\alpha}$ holds.

Conversely, suppose that $(a_{mn})\in \{B(\mathcal{M}_{u}) \} ^{\alpha}\setminus\mathcal{L}_{u}$. Then we have $\sum_{m,n}|a_{mn}x_{mn}|<\infty$ for all $x=(x_{mn})\in B(\mathcal {M}_{u})$. We can easily say with the special case $x=(x_{mn})=\{ (-1)^{m+n}\}\in B(\mathcal{M}_{u})$ that

$$ \sum_{m,n}|a_{mn}x_{mn}|=\sum _{m,n}|a_{mn}|=\infty. $$

This means that $(a_{mn})\notin \{B(\mathcal{M}_{u}) \} ^{\alpha}$, which contradicts the hypothesis. Therefore, $(a_{mn})$ must belong to the space $\in\mathcal{L}_{u}$.

Since the proof can be given for the space $B(\mathcal{C}_{bp})$ in a similar way, we omit the details. □

The *α*- and *γ*-duals of a double sequence space are unique. But $\beta(\vartheta)$-dual of a double sequence space can be more than one according to the *ϑ*-convergence. In this part, we give the $\beta(\vartheta)$- and *γ*-duals of the new double sequence spaces. The conditions for the characterization of the four-dimensional matrices transformed the spaces $\mathcal{C}_{bp}$, $\mathcal{C}_{r}$ and $\mathcal{C}_{p}$ into the space $\mathcal {C}_{bp}$ are well known (see [9, 10] and [5]).

### Lemma 3.2

*A four*-*dimensional matrix* $A=(a_{mnkl})\in(\mathcal{C}_{bp}:\mathcal {C}_{\vartheta})$ *if and only if the following conditions hold*:

3.1 $$\begin{aligned}& \sup_{m,n\in\mathbb{N}}\sum_{k,l}|a_{mnkl}|< \infty , \end{aligned}$$

3.2 $$\begin{aligned}& \exists a_{kl}\in\mathbb{C} \ni \vartheta\mbox{-} \lim_{m,n\to\infty}a_{mnkl}=a_{kl}\quad \textit{for all } k,l\in\mathbb {N}, \end{aligned}$$

3.3 $$\begin{aligned}& \exists l\in\mathbb{C} \ni \vartheta\mbox{-} \lim _{m,n\to \infty}\sum_{k,l}a_{mnkl}=l \quad \textit{exists}, \end{aligned}$$

3.4 $$\begin{aligned}& \exists k_{0}\in\mathbb{N}\ni\vartheta\mbox{-} \lim_{m,n\to \infty}\sum_{l}|a_{mnk_{0}l}-a_{k_{0}l}|=0, \end{aligned}$$

3.5 $$\begin{aligned}& \exists l_{0}\in\mathbb{N}\ni\vartheta\mbox{-} \lim_{m,n\to \infty}\sum_{k}|a_{mnkl_{0}}-a_{kl_{0}}|=0. \end{aligned}$$

*In the case* (3.5), $a=(a_{kl})\in\mathcal{L}_{u}$ *and*

$$ \vartheta\mbox{-} \lim_{m,n\to\infty}[Ax]_{m,n}=\sum _{k,l}a_{kl}x_{kl}+ \biggl(l \mbox{-} \sum_{k,l}a_{kl} \biggr)bp \mbox{-} \lim_{m,n\to \infty}x_{mn} $$

*holds for* $x\in\mathcal{C}_{bp}$

### Lemma 3.3

*A four*-*dimensional matrix* $A=(a_{mnkl})\in(\mathcal{C}_{p}:\mathcal {C}_{\vartheta})$ *if and only if* (3.1)-(3.3) *hold and the following conditions also hold*:

3.6 $$\begin{aligned}& \forall k\in\mathbb{N}, \exists l_{0}\in \mathbb{N} \ni a_{mnkl}=0 \quad \textit{for all } l>l_{0} \textit{ and } m,n\in \mathbb{N}, \end{aligned}$$

3.7 $$\begin{aligned}& \forall l\in\mathbb{N}, \exists k_{0}\in \mathbb{N} \ni a_{mnkl}=0\quad \textit{for all } k>k_{0} \textit{ and } m,n\in \mathbb{N}. \end{aligned}$$

*In the case* (3.7) $\exists k_{0}, l_{0}\in\mathbb{N}$ *such that* $a=(a_{kl})\in\mathcal{L}_{u}$ *and* $(a_{kl_{0}})_{k\in\mathbb{N}}, (a_{k_{0}l})_{l\in\mathbb{N}} \in\varphi$, *where* *φ* *denotes the spaces of all finitely non*-*zero sequences and*

$$ \vartheta\mbox{-} \lim_{m,n\to\infty}[Ax]_{m,n}=\sum _{k,l}a_{kl}x_{kl}+\sum _{k} \biggl(L\mbox{-} \sum _{k,l}a_{kl} \biggr)p\mbox{-} \lim _{m,n\to\infty}x_{mn} $$

*holds for* $x=(x_{kl})\in\mathcal{C}_{p}$.

### Lemma 3.4

*A four*-*dimensional matrix* $A=(a_{mnkl})\in(\mathcal{C}_{r}:\mathcal {C}_{\vartheta})$ *if and only if* (3.1)-(3.3) *hold and the following conditions also hold*:

3.8 $$\begin{aligned}& \exists l_{0}\in\mathbb{N} \ni\vartheta\mbox{-} \lim_{m,n\to \infty}\sum_{k}a_{mnkl_{0}}=u_{l_{0}}, \end{aligned}$$

3.9 $$\begin{aligned}& \exists k_{0}\in\mathbb{N} \ni\vartheta\mbox{-} \lim_{m,n\to \infty}\sum_{l}a_{mnk_{0}l}=v_{k_{0}}. \end{aligned}$$

*In the case* (3.9), $a=(a_{kl})\in\mathcal{L}_{u}$ *and* $(u_{l}), (v_{k})\in\ell_{1}$ *and*

$$\begin{aligned} \vartheta\mbox{-} \lim_{m,n\to\infty}[Ax]_{m,n} =& \sum_{k,l}a_{kl}x_{kl}+\sum _{k} \biggl(v_{k}\mbox{-} \sum _{l}a_{kl} \biggr)x_{k}+\sum _{l} \biggl(u_{l}\mbox{-} \sum _{k}a_{kl} \biggr)x_{l} \\ &{}+ \biggl(L+\sum_{k,l}a_{kl}\mbox{-} \sum_{k}v_{k}\mbox{-} \sum_{l}u_{l} \biggr)r\mbox{-} \lim_{m,n\to\infty}x_{mn} \end{aligned}$$

*holds for* $x\in\mathcal{C}_{r}$.

### Theorem 3.5

*A four*-*dimensional matrix* $A=(a_{mnkl})\in(\mathcal{C}_{bp}: \mathcal {M}_{u})$ *if and only if* (3.1) *holds*.

### Proof

Let the four-dimensional matrix $A=(a_{mnkl})\in(\mathcal{C}_{bp}: \mathcal{M}_{u})$. Then *Ax* exists and is in $\mathcal{M}_{u}$ for all $x=(x_{kl})\in\mathcal{C}_{bp}$. That is, $A_{mn}\in\mathcal{M}_{u}$ for each $m,n\in\mathbb{N}$. Therefore,

$$\begin{aligned} \|Ax\|_{\infty} =&\sup_{m,n\in\mathbb{N}} \biggl\vert \sum _{k,l}a_{mnkl}x_{kl} \biggr\vert \\ \leq&\sup_{m,n\in\mathbb{N}}\sum_{k,l}|a_{mnkl}||x_{kl}|< \infty. \end{aligned}$$

Then condition (3.1) is sufficient.

Conversely, suppose that condition (3.1) is satisfied for all $x=(x_{kl})\in\mathcal{C}_{bp}$. Then

$$ \biggl\vert \sum_{k,l}a_{mnkl}x_{kl} \biggr\vert \leq\sum_{k,l}|a_{mnkl}||x_{kl}|. $$

We have, after taking supremum over $m,n\in\mathbb{N}$, that

$$ \sup_{m,n\in\mathbb{N}} \biggl\vert \sum_{k,l}a_{mnkl}x_{kl} \biggr\vert \leq \sup_{m,n\in\mathbb{N}}\sum_{k,l}|a_{mnkl}|M< \infty. $$

Then it is derived from the last approaches that $Ax\in\mathcal {M}_{u}$. This completes the proof. □

### Lemma 3.6

[11]

*Let* $A=(a_{mnkl})$ *be a four*-*dimensional matrix*. *Then the following statements hold*:

(i) *For* $0< q\leq1$, $A\in(\mathcal{L}_{q}:\mathcal{M}_{u})$ *if and only if*
  3.10 $$ \sup_{m,n,k,l\in\mathbb{N}}|a_{mnkl}|< \infty. $$
(ii) *For* $1< q<\infty$, $A\in(\mathcal{L}_{q}:\mathcal{M}_{u})$ *if and only if*
  3.11 $$ \sup_{m,n\in\mathbb{N}}\sum_{k,l}|a_{mnkl}|^{q'}< \infty, \quad \textit{where } \frac{1}{q}+\frac{1}{q'}=1. $$

### Lemma 3.7

[11]

*Let* $A=(a_{mnkl})$ *be a four*-*dimensional matrix*. *Then the following statements hold*:

(i) *For* $0< q\leq1$, $A\in(\mathcal{L}_{q}:\mathcal{C}_{bp})$ *if and only if conditions* (3.2) *and* (3.10) *hold with* $\vartheta=bp$.
(ii) *For* $1< q<\infty$, $A\in(\mathcal{L}_{q}:\mathcal {C}_{bp})$ *if and only if conditions* (3.2) *and* (3.11) *hold with* $\vartheta=bp$.

### Lemma 3.8

[12]

*A four*-*dimensional matrix* $A=(a_{mnkl})\in(\mathcal{M}_{u}:\mathcal {C}_{bp})$ *if and only if conditions* (3.1)-(3.2) *hold and the following conditions also hold*:

3.12 $$\begin{aligned}& \exists a_{kl}\in\mathbb{C}\ni bp\mbox{-} \lim _{m,n\to \infty}\sum_{k,l}|a_{mnkl}-a_{kl}|=0, \end{aligned}$$

3.13 $$\begin{aligned}& bp\mbox{-} \lim_{m,n\to\infty}\sum _{l=0}^{n} a_{mnkl} \quad \textit{exists for each } k\in\mathbb{N}, \end{aligned}$$

3.14 $$\begin{aligned}& bp\mbox{-} \lim_{m,n\to\infty}\sum _{k=0}^{m} a_{mnkl} \quad \textit{exists for each } l\in\mathbb{N}, \end{aligned}$$

3.15 $$\begin{aligned}& \sum_{k,l}|a_{mnkl}|\quad \textit{converges}. \end{aligned}$$

### Lemma 3.9

[13]

*A four*-*dimensional matrix* $A=(a_{mnkl})\in(\mathcal{M}_{u}:\mathcal {M}_{u})$ *if and only if condition* (3.1) *holds*.

### Lemma 3.10

[14]

*A four*-*dimensional matrix* $A=(a_{mnkl})\in(\mathcal{M}_{u}:\mathcal {C}_{p})$ *if and only if conditions* (3.2), (3.6) *and* (3.7) *hold*.

Let us define the sets $d_{k}(r,s,t,u)$ with $k\in\{1,2,\ldots, 14\}$ as follows:

$$\begin{aligned}& d_{1}(r,s,t,u)= \Biggl\{ a=(a_{kl})\in\Omega: \sup _{m,n\in\mathbb {N}}\sum_{k,l} \Biggl\vert \sum _{j,i=k,l}^{m,n} \biggl(\frac{-s}{r} \biggr)^{j-k} \biggl(\frac{-u}{t} \biggr)^{i-l} \frac{a_{ji}}{rt} \Biggr\vert ^{q'}< \infty \Biggr\} , \\& d_{2}(r,s,t,u)= \Biggl\{ a=(a_{kl})\in\Omega: \exists \beta_{kl}\in \mathbb{C}\ni\vartheta\mbox{-} \lim _{m,n\to\infty}\sum_{j,i=k,l}^{m,n} \biggl(\frac{-s}{r} \biggr)^{j-k} \biggl(\frac {-u}{t} \biggr)^{i-l}a_{ji}=\beta_{kl} \Biggr\} , \\& d_{3}(r,s,t,u)= \Biggl\{ a=(a_{kl})\in\Omega: \\& \hphantom{d_{3}(r,s,t,u)={}}\exists l\in \mathbb {C}\ni \vartheta\mbox{-} \lim_{m,n\to\infty}\sum _{k,l}\sum_{j,i=k,l}^{m,n} \biggl(\frac{-s}{r} \biggr)^{j-k} \biggl(\frac {-u}{t} \biggr)^{i-l}\frac{a_{ji}}{rt}=l\text{ exists} \Biggr\} , \\ & d_{4}(r,s,t,u)= \Biggl\{ a=(a_{kl})\in\Omega: \\ & \hphantom{d_{4}(r,s,t,u)={}}\exists l_{0}\in\mathbb {N}\ni\vartheta\mbox{-} \lim _{m,n\to\infty}\sum_{k} \Biggl\vert \sum _{j,i=k,l_{0}}^{m,n} \biggl(\frac{-s}{r} \biggr)^{j-k} \biggl(\frac {-u}{t} \biggr)^{i-l_{0}}a_{ji}- \beta_{kl_{0}} \Biggr\vert =0 \Biggr\} , \\ & d_{5}(r,s,t,u)= \Biggl\{ a=(a_{kl})\in\Omega: \\ & \hphantom{d_{5}(r,s,t,u)= {}}\exists k_{0}\in\mathbb {N}\ni\vartheta\mbox{-} \lim _{m,n\to\infty}\sum_{l} \Biggl\vert \sum _{j,i=k_{0},l}^{m,n} \biggl(\frac{-s}{r} \biggr)^{j-k_{0}} \biggl(\frac {-u}{t} \biggr)^{i-l}a_{ji}- \beta_{k_{0}l} \Biggr\vert =0 \Biggr\} , \\ & d_{6}(r,s,t,u)= \Biggl\{ a=(a_{kl})\in\Omega: \\ & \hphantom{d_{6}(r,s,t,u)={}}\forall k\in \mathbb{N}, \exists l_{0}\in\mathbb{N}\ni\sum _{j,i=k,l}^{m,n} \biggl(\frac {-s}{r} \biggr)^{j-k} \biggl(\frac{-u}{t} \biggr)^{i-l} \frac {a_{ji}}{rt}=0 \ \forall l>l_{0} \text{ and }\forall m,n\in \mathbb{N} \Biggr\} , \\ & d_{7}(r,s,t,u)= \Biggl\{ a=(a_{kl})\in\Omega: \\ & \hphantom{d_{7}(r,s,t,u)={}}\forall l\in \mathbb{N}, \exists k_{0}\in\mathbb{N}\ni\sum _{j,i=k,l}^{m,n} \biggl(\frac {-s}{r} \biggr)^{j-k} \biggl(\frac{-u}{t} \biggr)^{i-l} \frac {a_{ji}}{rt}=0 \ \forall k>k_{0} \text{ and }\forall m,n\in \mathbb{N} \Biggr\} , \\ & d_{8}(r,s,t,u)= \Biggl\{ a=(a_{kl})\in\Omega: \sup _{m,n\in\mathbb {N}} \Biggl\vert \sum_{j,i=k,l}^{m,n} \biggl(\frac{-s}{r} \biggr)^{j-k} \biggl(\frac{-u}{t} \biggr)^{i-l}\frac{a_{ji}}{rt} \Biggr\vert ^{q'}< \infty \Biggr\} , \\ & d_{9}(r,s,t,u)= \Biggl\{ a=(a_{kl})\in\Omega: \\ & \hphantom{d_{9}(r,s,t,u)={}}\exists l_{0}\in\mathbb {N}\ni\vartheta\mbox{-} \lim _{m,n\to\infty}\sum_{k}\sum _{j,i=k,l_{0}}^{m,n} \biggl(\frac{-s}{r} \biggr)^{j-k} \biggl(\frac {-u}{t} \biggr)^{i-l_{0}} \frac{a_{ji}}{rt}=u_{l_{0}} \Biggr\} , \\ & d_{10}(r,s,t,u)= \Biggl\{ a=(a_{kl})\in\Omega: \\ & \hphantom{d_{10}(r,s,t,u)={}}\exists k_{0}\in\mathbb {N}\ni\vartheta\mbox{-} \lim _{m,n\to\infty}\sum_{l}\sum _{j,i=k_{0},l}^{m,n} \biggl(\frac{-s}{r} \biggr)^{j-k_{0}} \biggl(\frac {-u}{t} \biggr)^{i-l} \frac{a_{ji}}{rt}=v_{k_{0}} \Biggr\} , \\ & d_{11}(r,s,t,u)= \Biggl\{ a=(a_{kl})\in\Omega: \\ & \hphantom{d_{11}(r,s,t,u)={}}\exists \beta_{kl}\in \mathbb{C}\ni\vartheta\mbox{-} \lim _{m,n\to\infty}\sum_{k,l} \Biggl\vert \sum _{j,i=k,l}^{m,n} \biggl(\frac{-s}{r} \biggr)^{j-k} \biggl(\frac {-u}{t} \biggr)^{i-l} \frac{a_{ji}}{rt}-\beta_{kl} \Biggr\vert =0 \Biggr\} , \\ & d_{12}(r,s,t,u)= \Biggl\{ a=(a_{kl})\in\Omega: \\& \hphantom{d_{12}(r,s,t,u)={}}\forall k\in \mathbb {N}, \vartheta\mbox{-} \lim_{m,n\to\infty}\sum _{l=0}^{n} \sum_{j,i=k,l_{0}}^{m,n} \biggl(\frac{-s}{r} \biggr)^{j-k} \biggl(\frac {-u}{t} \biggr)^{i-l_{0}}\frac{a_{ji}}{rt} \text{ exists} \Biggr\} , \\& d_{13}(r,s,t,u)= \Biggl\{ a=(a_{kl})\in\Omega: \\& \hphantom{d_{13}(r,s,t,u)={}}\forall l\in \mathbb {N}, \vartheta\mbox{-} \lim_{m,n\to\infty}\sum _{k=0}^{m} \sum_{j,i=k,l_{0}}^{m,n} \biggl(\frac{-s}{r} \biggr)^{j-k} \biggl(\frac {-u}{t} \biggr)^{i-l_{0}}\frac{a_{ji}}{rt} \text{ exists} \Biggr\} , \\& d_{14}(r,s,t,u)= \Biggl\{ a=(a_{kl})\in\Omega:\sum _{k,l} \Biggl\vert \sum_{j,i=k,l_{0}}^{m,n} \biggl(\frac{-s}{r} \biggr)^{j-k} \biggl(\frac {-u}{t} \biggr)^{i-l_{0}}\frac{a_{ji}}{rt} \Biggr\vert \text{ converges} \Biggr\} . \end{aligned}$$

### Theorem 3.11

*The following statements hold*:

(i) $\{B(\mathcal{M}_{u}) \}^{\gamma}=d_{1}(r,s,t,u)$ *with* $q'=1$.
(ii) $\{B(\mathcal{L}_{q}) \}^{\gamma}=\bigl \{ \scriptsize{ \begin{array}{l@{\quad}l} d_{1}(r,s,t,u), & 1\leq q<\infty; \\ d_{8}(r,s,t,u), & 0< q<1. \end{array}} \bigr.$
(iii) $\{B(\mathcal{C}_{bp}) \}^{\gamma}=d_{1}(r,s,t,u)$ *with* $q'=1$.

### Proof

(iii) Let us suppose that $a=(a_{mn})\in\Omega$ and $x=(x_{mn})\in B(\mathcal{C}_{bp})$. Then we have $y=Bx\in\mathcal{C}_{bp}$. Therefore, we have the following equality for the $m,n$th partial sum of $\sum_{k,l}a_{kl}x_{kl}$:

3.16 $$\begin{aligned} \sum_{k,l=0}^{m,n}a_{kl}x_{kl} =& \sum_{k,l=0}^{m,n}a_{kl}\sum _{j,i=0}^{m,n} \biggl(\frac{-s}{r} \biggr)^{k-j} \biggl(\frac{-u}{t} \biggr)^{l-i} \frac{y_{ji}}{rt} \\ =&\sum_{k,l=0}^{m,n}\sum _{j,i=k,l}^{m,n} \biggl(\frac{-s}{r} \biggr)^{j-k} \biggl(\frac{-u}{t} \biggr)^{i-l} \frac{a_{ji}}{rt}y_{kl} \\ =&(Dy)_{mn}, \end{aligned}$$

where the four-dimensional matrix $D=(d_{mnkl})$ is defined by

$$ d_{mnkl}= \textstyle\begin{cases} \sum_{j,i=k,l}^{m,n} (\frac{-s}{r} )^{j-k} (\frac {-u}{t} )^{i-l}\frac{a_{ji}}{rt}, & 0\leq k\leq m, 0\leq l\leq n; \\ 0, & \text{otherwise} \end{cases} $$

for all $k,k,m,n\in\mathbb{N}$. Then we can say that $ax\in\mathcal {BS}$ whenever $x=(x_{mn})\in B(\mathcal{C}_{bp})$ if and only if $Dy\in\mathcal{M}_{u}$ whenever $y=(y_{mn})\in\mathcal{C}_{bp}$. This means that $a=(a_{mn})\in \{B(\mathcal{C}_{bp}) \}^{\gamma } $ if and only if $D\in(\mathcal{C}_{bp}: \mathcal{M}_{u})$. Thus, one can easily see that the conditions of Theorem 3.5 hold, that is,

$$ \sup_{m,n\in\mathbb{N}}\sum_{k,l} \Biggl\vert \sum_{j,i=k,l}^{m,n} \biggl(\frac{-s}{r} \biggr)^{j-k} \biggl(\frac{-u}{t} \biggr)^{i-l} \frac {a_{ji}}{rt} \Biggr\vert < \infty, $$

which is the set $d_{1}(r,s,t,u)$ with $q'=1$. This completes the proof of Part (iii).

The proofs of Parts (i) and (ii) can be shown in a similar way by using Lemmas 3.9 and 3.6, respectively, instead of Lemma 3.5. Thus, we omit the details. □

### Theorem 3.12

*The following statements hold*:

(i) $\{B(\mathcal{C}_{bp}) \}^{\beta(\vartheta )}=\bigcap_{i=1}^{5}d_{i}(r,s,t,u)$ *with* $q'=1$.
(ii) $\{B(\mathcal{C}_{p}) \}^{\beta(\vartheta )}=\bigcap_{i=1}^{3}d_{i}(r,s,t,u)\cap d_{6}(r,s,t,u)\cap d_{7}(r,s,t,u)$ *with* $q'=1$.
(iii) $\{B(\mathcal{C}_{r}) \}^{\beta(\vartheta )}=\bigcap_{i=1}^{3}d_{i}(r,s,t,u)\cap d_{9}(r,s,t,u)\cap d_{10}(r,s,t,u)$ *with* $q'=1$.
(iv) $\{B(\mathcal{L}_{q}) \}^{\beta (bp)}=d_{1}(r,s,t,u)\cap d_{2}(r,s,t,u)$ *for* $1< q<\infty$.
(v) $\{B(\mathcal{L}_{q}) \}^{\beta (bp)}=d_{2}(r,s,t,u)\cap d_{8}(r,s,t,u)$ *with* $q'=1$ *for* $0< q\leq1$.
(vi) $\{B(\mathcal{M}_{u}) \}^{\beta (bp)}=d_{1}(r,s,t,u)\cap d_{2}(r,s,t,u)\bigcap_{i=11}^{14}d_{i}(r,s,t,u)$.
(vii) $\{B(\mathcal{M}_{u}) \}^{\beta (p)}=d_{2}(r,s,t,u)\cap d_{6}(r,s,t,u)\cap d_{7}(r,s,t,u)$.

### Proof

Suppose that $a=(a_{mn})\in\Omega$ and $x=(x_{mn})\in B(\mathcal {C}_{bp})$. Then there exists a sequence $y=(y_{mn})\in\mathcal {C}_{bp}$ with $Bx=y$. Therefore, since (3.16) holds, one can conclude that $ax\in\mathcal{CS}_{\vartheta}$ whenever $x=(x_{mn})\in B(\mathcal{C}_{bp})$ if and only if $Dy\in\mathcal {C}_{\vartheta}$ whenever $y=(y_{mn})\in\mathcal{C}_{bp}$. It gives us that $a=(a_{mn})\in \{B(\mathcal{C}_{bp}) \}^{\beta (\vartheta)} $ if and only if $D\in(\mathcal{C}_{bp}:\mathcal {C}_{\vartheta})$. Hence, the conditions of Lemma 3.2 are satisfied with $d_{mnkl}$ instead of $a_{mnkl}$. That is,

$$\begin{aligned}& \sup_{m,n\in\mathbb{N}}\sum_{k,l}|d_{mnkl}|< \infty, \\& \exists\beta_{kl}\in\mathbb{C} \ni \vartheta\mbox{-} \lim _{m,n\to \infty}d_{mnkl}=\beta_{kl}\quad \text{for all } k,l\in\mathbb{N}, \\& \exists l\in\mathbb{C} \ni \vartheta\mbox{-} \lim_{m,n\to\infty} \sum_{k,l}d_{mnkl}=l\quad \text{exists}, \\& \exists k_{0}\in\mathbb{N}\ni\vartheta\mbox{-} \lim _{m,n\to\infty}\sum_{l}|d_{mnk_{0}l}- \beta_{k_{0}l}|=0, \\& \exists l_{0}\in\mathbb{N}\ni\vartheta\mbox{-} \lim _{m,n\to\infty}\sum_{k}|d_{mnkl_{0}}- \beta_{kl_{0}}|=0, \end{aligned}$$

which give the $\beta(\vartheta)$-dual of the space $B(\mathcal {C}_{bp})$ is $\bigcap_{i=1}^{5}d_{i}(r,s,t,u)$. This completes the proof of Part (i). Since Parts (ii)-(vii) can be proved in a similar way by using Lemmas 3.3, 3.4, 3.7, 3.8 and 3.10, respectively, to avoid the repetition of similar statements, we omit their proofs. □

## Characterization of some classes of four-dimensional matrices

In this section, we characterize some four-dimensional matrix classes which are related to the double sequence spaces derived as the domain of the four-dimensional generalized difference matrix in the spaces $\mathcal{M}_{u}$, $\mathcal{C}_{p}$, $\mathcal{C}_{bp}$, $\mathcal{C}_{r}$ and $\mathcal{L}_{q}$ by using the concept of four-dimensional dual summability methods for double sequences introduced and studied by Başar [15] and Yeşilkayagil and Başar [16].

Now, let us suppose that the four-dimensional matrices $A=(a_{mnkl})$ and $E=(e_{mnkl})$ transform the sequences $x=(x_{mn})$ and $y=(y_{mn})$ which are connected with relation (1.2) to the double sequences $s=(s_{mn})$ and $z=(z_{mn})$, respectively, that is,

4.1 $$\begin{aligned}& s_{mn}=(Ax)_{mn}=\sum _{k,l=0}^{\infty}a_{mnkl}x_{kl}\quad \text{for all }m,n\in\mathbb{N}, \end{aligned}$$

4.2 $$\begin{aligned}& z_{mn}=(Ey)_{mn}=\sum _{k,l=0}^{\infty}e_{mnkl}y_{kl}\quad \text{for all }m,n\in\mathbb{N}. \end{aligned}$$

It is obvious that the method *B* is applied to the $B(r,s,t,u)$-transform of the sequence *x*, while the method *A* is directly applied to the elements of the sequence *x*. Then we can say that the methods *A* and *E* are essentially different.

Let us assume that the usual matrix product $EB(r,s,t,u)$ exists, which is a much weaker hypothesis than the conditions on the matrix *E* belonging to any class of matrices, in general. We can say in this case that the matrices *A* and *E* in (4.1) and (4.2) are the dual summability methods if *s* is reduced to *z* or viceversa under the application of the usual summation by parts. This leads us to the fact that $EB(r,s,t,u)$ exists and is equal to *A*, and $Ax=\{ EB(r,s,t,u)x\}=E\{B(r,s,t,u)x\}=Ey$ formally holds if one side exists. This statement is equivalent to the relation between the elements of the matrices $A=(a_{mnkl})$ and $E=(e_{mnkl})$

4.3 $$ \begin{aligned} &a_{mnkl}=sue_{mn,m-1,n-1}+ste_{mn,m-1,n}+rue_{mnm,n-1}+rte_{mnmn} \quad \text{or equivalently} \\ &e_{mnkl}=\sum_{i,j=k,l}^{\infty} \biggl( \frac{-s}{r} \biggr)^{i-k} \biggl(\frac{-u}{t} \biggr)^{j-l}\frac{a_{mnij}}{rt} \end{aligned} $$

for all $m,n,k,l\in\mathbb{N}$. It is trivial that relation (4.3) between the elements of the matrices $A=(a_{mnkl})$ and $E=(e_{mnkl})$ can be stated by the matrix product as follows:

$$ A=EB(r,s,t,u)\quad \text{or equivalently}\quad E=AF(r,s,t,u). $$

For the sake of brevity in notation, we may also write here and after for all $m,n,k,l\in\mathbb{N}$ that

4.4 $$ e(m,n)=\sum_{k,l=0}^{m,n}\sum _{i,j=k,l}^{\infty} \biggl(\frac {-s}{r} \biggr)^{i-k} \biggl(\frac{-u}{t} \biggr)^{j-l} \frac{a_{mnij}}{rt} $$

and

$$\begin{aligned}& \Delta_{10}^{kl}a_{mnkl}=a_{mnkl}-a_{mn,k+1,l}, \\& \Delta_{01}^{kl}a_{mnkl}=a_{mnkl}-a_{mnk,l+1}, \\& \Delta_{11}^{kl}a_{mnkl}=\Delta_{10}^{kl} \bigl(\Delta _{01}^{kl}a_{mnkl}\bigr)= \Delta_{01}^{kl}\bigl(\Delta_{10}^{kl}a_{mnkl} \bigr). \end{aligned}$$

Now, we may give the following theorem by using equality (4.3) between the methods *A* and *E*.

### Theorem 4.1

*Suppose that the elements of four*-*dimensional infinite matrices* $A=(a_{mnkl})$ *and* $E=(e_{mnkl})$ *are connected with relation* (4.3). *Then* $A\in(B(\lambda):\mu)$ *if and only if* $A_{mn}\in [B(\lambda)]^{\beta(\vartheta)}$ *for all* $m,n\in\mathbb{N}$ *and* $E\in(\lambda:\mu)$, *where* $\lambda,\mu\in \{\mathcal{M}_{u}, \mathcal{C}_{p}, \mathcal{C}_{bp}, \mathcal{C}_{r}, \mathcal{L}_{q} \}$.

### Proof

Suppose that $A\in(B(\lambda):\mu)$. Then *Ax* exists and is in *μ* for all $x=(x_{mn})\in B(\lambda)$, which implies the fact that $A_{mn}\in[B(\lambda)]^{\beta(\vartheta)}$ for all $m,n\in\mathbb {N}$. Thus, we have the following equality derived from the partial sum of the series $\sum_{k,l}a_{mnkl}x_{kl}$ with relations (4.3):

4.5 $$ \sum_{k,l=0}^{m,n}a_{mnkl}x_{kl}= \sum_{k,l=0}^{m,n} \Biggl[\sum _{i,j=k,l}^{m,n} \biggl(\frac{-s}{r} \biggr)^{i-k} \biggl(\frac {-u}{t} \biggr)^{j-l} \frac{a_{mnij}}{rt} \Biggr]y_{kl} $$

for all $m,n\in\mathbb{N}$. Then, by taking *ϑ*-limit on (4.5) as $m,n\to\infty$, we have $Ax=Ey$. Hence, $Ey\in\mu $ whenever $y\in\lambda$, i.e., $E\in(\lambda:\mu)$.

Conversely, suppose that $A_{mn}\in[B(\lambda)]^{\beta(\vartheta)}$ for all $m,n\in\mathbb{N}$ and $E\in(\lambda:\mu)$, and let $v=(v_{kl})\in B(\lambda)$ with $u=Bv$. Then *Av* exists. Therefore, one can derive from the $(\xi,\varrho)$th rectangular partial sum of the series $\sum_{k,l}a_{mnkl}v_{kl}$ for all $m,n,\xi,\varrho\in \mathbb{N}$ that

$$ \sum_{k,l=0}^{\xi,\varrho}a_{mnkl}v_{kl}= \sum_{k,l=0}^{\xi,\varrho }a_{mnkl}\sum _{i,j=0}^{k,l}f_{klij}u_{ij}=\sum _{k,l=0}^{\xi,\varrho } \Biggl(\sum _{i,j=k,l}^{\xi,\varrho} a_{mnij}f_{ijkl} \Biggr)u_{kl}, $$

which gives by letting *p*-limit as $\xi,\varrho\to\infty$ that

$$ \sum_{k,l}a_{mnkl}v_{kl}=\sum _{k,l}e_{mnkl}u_{kl} \quad \text{for all } m,n\in\mathbb{N}. $$

That is, $Av=Eu$, which leads to the fact $A\in(B(\lambda):\mu)$, as desired. □

By changing the role of the spaces $B(\lambda)$ and *μ* in Theorem 4.1, we have the following lemma.

### Lemma 4.2

[8], Theorem 4.7

*Let* *λ* *and* *μ* *be as in Theorem* 4.1, *and let the elements of the four*-*dimensional matrices* $A=(a_{mnkl})$ *and* $G=(g_{mnkl})$ *be connected with the relation*

4.6 $$ g_{mnkl}=\sum_{i,j=0}^{m,n}b_{mnij}(r,s,t,u)a_{ijkl} \quad \textit{for all } m,n,k,l\in\mathbb{N}. $$

*Then* $A\in(\mu:B(\lambda))$ *if and only if* $G\in(\mu:\lambda)$.

### Corollary 4.3

*Let* $A=(a_{mnkl})$ *be a four*-*dimensional infinite matrix*. *Then the following statements hold*.

(i) $A\in(B(\mathcal{C}_{p}):\mathcal{C}_{\vartheta})$ *if and only if* (3.1)-(3.3), (3.6) *and* (3.7) *hold with* $e_{mnkl}$ *instead of* $a_{mnkl}$.
(ii) $A\in(B(\mathcal{C}_{bp}):\mathcal{C}_{\vartheta})$ *if and only if* (3.1)-(3.3), (3.4) *and* (3.5) *hold with* $e_{mnkl}$ *instead of* $a_{mnkl}$.
(iii) $A\in(B(\mathcal{C}_{r}):\mathcal{C}_{\vartheta})$ *if and only if* (3.1)-(3.3), (3.8) *and* (3.9) *hold with* $e_{mnkl}$ *instead of* $a_{mnkl}$.
(iv) $A\in(B(\mathcal{L}_{q}):\mathcal{C}_{bp})$ *if and only if* (3.2) *and* (3.11) *hold for* $1< q<\infty$ *with* $e_{mnkl}$ *instead of* $a_{mnkl}$.
(v) $A\in(B(\mathcal{L}_{q}):\mathcal{C}_{bp})$ *if and only if* (3.2) *and* (3.10) *hold for* $0< q\leq1$ *with* $e_{mnkl}$ *instead of* $a_{mnkl}$.
(vi) $A\in(B(\mathcal{L}_{q}):\mathcal{M}_{u})$ *if and only if* (3.10) *holds for* $0< q<1$ *with* $e_{mnkl}$ *instead of* $a_{mnkl}$.
(vii) $A\in(B(\mathcal{L}_{q}):\mathcal{M}_{u})$ *if and only if* (3.11) *holds for* $1< q<\infty$ *with* $e_{mnkl}$ *instead of* $a_{mnkl}$.
(viii) $A\in(B(\mathcal{M}_{u}):\mathcal{C}_{bp})$ *if and only if* (3.1), (3.3), (3.12), (3.13),(3.14) *and* (3.15) *hold with* $e_{mnkl}$ *instead of* $a_{mnkl}$.
(ix) $A\in(B(\mathcal{M}_{u}):\mathcal{C}_{p})$ *if and only if* (3.2), (3.6) *and* (3.7) *hold with* $e_{mnkl}$ *instead of* $a_{mnkl}$.
(x) $A\in(B(\mathcal{C}_{bp}):\mathcal{M}_{u})$ *if and only if* (3.1) *holds with* $e_{mnkl}$ *instead of* $a_{mnkl}$.

### Corollary 4.4

*Let* $E=(e_{mnkl})$ *be a four*-*dimensional infinite matrix*. *Then the following statements hold*.

(i) $A\in(\mathcal{C}_{p}:B(\mathcal{C}_{\vartheta}))$ *if and only if* (3.1)-(3.3), (3.6) *and* (3.7) *hold with* $g_{mnkl}$ *instead of* $a_{mnkl}$.
(ii) $A\in(\mathcal{C}_{bp}:B(\mathcal{C}_{\vartheta}))$ *if and only if* (3.1)-(3.3), (3.4) *and* (3.5) *hold with* $g_{mnkl}$ *instead of* $a_{mnkl}$.
(iii) $A\in(\mathcal{C}_{r}:B(\mathcal{C}_{\vartheta}))$ *if and only if* (3.1)-(3.3), (3.8) *and* (3.9) *hold with* $g_{mnkl}$ *instead of* $a_{mnkl}$.
(iv) $A\in(\mathcal{L}_{q}:B(\mathcal{C}_{bp}))$ *if and only if* (3.2) *and* (3.10) *hold for* $0< q\leq1$ *with* $g_{mnkl}$ *instead of* $a_{mnkl}$.
(v) $A\in(\mathcal{L}_{q}:B(\mathcal{C}_{bp}))$ *if and only if* (3.2) *and* (3.11) *hold for* $1< q<\infty$ *with* $g_{mnkl}$ *instead of* $a_{mnkl}$.
(vi) $A\in(\mathcal{L}_{q}:B(\mathcal{M}_{u}))$ *if and only if* (3.10) *holds for* $0< q<\leq1$ *with* $g_{mnkl}$ *instead of* $a_{mnkl}$.
(vii) $A\in(\mathcal{L}_{q}:B(\mathcal{M}_{u}))$ *if and only if* (3.11) *holds for* $1< q<\infty$ *with* $g_{mnkl}$ *instead of* $a_{mnkl}$.
(viii) $A\in(\mathcal{M}_{u}:B(\mathcal{C}_{bp}))$ *if and only if* (3.1), (3.3), (3.12), (3.13),(3.14) *and* (3.15) *hold with* $g_{mnkl}$ *instead of* $a_{mnkl}$.
(ix) $A\in(\mathcal{M}_{u}:B(\mathcal{C}_{p}))$ *if and only if* (3.2), (3.6) *and* (3.7) *hold with* $g_{mnkl}$ *instead of* $a_{mnkl}$.
(x) $A\in(\mathcal{C}_{bp}:B(\mathcal{M}_{u}))$ *if and only if* (3.1) *holds with* $g_{mnkl}$ *instead of* $a_{mnkl}$.

### Theorem 4.5

*Suppose that the elements of the four*-*dimensional matrices* $A=(a_{mnkl})$ *and* $H=(h_{mnkl})$ *are connected with the relation*

4.7 $$ h_{mnkl}=\sum_{i,j=k,l}^{m,n}b_{mnij}(r,s,t,u)e_{ijkl} \quad \textit{for all }m,n,k,l\in\mathbb{N}, $$

*where the four*-*dimensional matrix* $E=(e_{mnkl})$ *is defined as in* (4.3). *Then* $A\in(B(\lambda):B(\mu))$ *if and only if* $H\in (\lambda:\mu)$, *where* $\lambda,\mu\in \{\mathcal{M}_{u}, \mathcal{C}_{p}, \mathcal{C}_{bp}, \mathcal{C}_{r}, \mathcal{L}_{q} \}$.

### Proof

Suppose that $A\in(B(\lambda):B(\mu))$. Then *Ax* exists and is in $B(\mu)$ for all $x=(x_{mn})\in B(\lambda)$ and $\{B(Ax)\}_{mn}\in \mu$ for all $m,n\in\mathbb{N}$. Furthermore, we can say that the relation $Bx=y\in\lambda$ implies $B(AB^{-1}y)\in\mu$. By using relations (4.7) between the matrices $A=(a_{mnkl})$ and $H=(H_{mnkl})$ and relation (1.3) between $x=(x_{mn})$ and $y=(y_{mn})$, we can write the following equality derived from the partial sum of the series $\sum_{kl}h_{mnkl}y_{kl}$:

4.8 $$ \sum_{k,l=0}^{m,n}h_{mnkl}y_{kl}= \sum_{k,l=0}^{m,n}\sum _{i,j=k,l}^{m,n}b_{mnij}(r,s,t,u)e_{ijkl}y_{kl} $$

for all $m,n,k,l\in\mathbb{N}$. When we apply the *ϑ*-limit on equality (4.8) as $m,n\to\infty$, we have $Ax=Hy$. So, $Hy\in\mu$ whenever $y\in\lambda$ says that $H\in(\lambda:\mu)$. This completes the proof. □

### Corollary 4.6

*Let* $A=(a_{mnkl})$ *be a four*-*dimensional infinite matrix*. *Then the following statements hold*.

(i) $A\in(B(\mathcal{C}_{p}):B(\mathcal{C}_{\vartheta}))$ *if and only if* (3.1)-(3.3), (3.6) *and* (3.7) *hold with* $h_{mnkl}$ *instead of* $a_{mnkl}$.
(ii) $A\in(B(\mathcal{C}_{bp}):B(\mathcal{C}_{\vartheta}))$ *if and only if* (3.1)-(3.3), (3.4) *and* (3.5) *hold with* $h_{mnkl}$ *instead of* $a_{mnkl}$.
(iii) $A\in(B(\mathcal{C}_{r}):B(\mathcal{C}_{\vartheta}))$ *if and only if* (3.1)-(3.3), (3.8) *and* (3.9) *hold with* $h_{mnkl}$ *instead of* $a_{mnkl}$.
(iv) $A\in(B(\mathcal{L}_{q}):B(\mathcal{C}_{bp}))$ *if and only if* (3.2) *and* (3.10) *hold for* $0< q\leq1$ *with* $h_{mnkl}$ *instead of* $a_{mnkl}$.
(v) $A\in(B(\mathcal{L}_{q}):B(\mathcal{C}_{bp}))$ *if and only if* (3.2) *and* (3.11) *hold for* $1< q<\infty$ *with* $h_{mnkl}$ *instead of* $a_{mnkl}$.
(vi) $A\in(B(\mathcal{L}_{q}):B(\mathcal{M}_{u}))$ *if and only if* (3.10) *holds for* $0< q\leq1$ *with* $h_{mnkl}$ *instead of* $a_{mnkl}$.
(vii) $A\in(B(\mathcal{L}_{q}):B(\mathcal{M}_{u}))$ *if and only if* (3.11) *holds for* $1< q<\infty$ *with* $h_{mnkl}$ *instead of* $a_{mnkl}$.
(viii) $A\in(B(\mathcal{M}_{u}):B(\mathcal{C}_{bp}))$ *if and only if* (3.1), (3.3), (3.12), (3.13),(3.14) *and* (3.15) *hold with* $h_{mnkl}$ *instead of* $a_{mnkl}$.
(ix) $A\in(B(\mathcal{M}_{u}):B(\mathcal{C}_{p}))$ *if and only if* (3.2), (3.6) *and* (3.7) *hold with* $h_{mnkl}$ *instead of* $a_{mnkl}$.
(x) $A\in(B(\mathcal{C}_{bp}):B(\mathcal{M}_{u}))$ *if and only if* (3.1) *holds with* $h_{mnkl}$ *instead of* $a_{mnkl}$.

### Corollary 4.7

*Let* $A=(a_{mnkl})$ *be a four*-*dimensional infinite matrix*. *Then the following statements hold*.

(i) $A\in(B(\mathcal{C}_{p}):\mathcal{CS}_{\vartheta})$ *if and only if* (3.1)-(3.3), (3.6) *and* (3.7) *hold with* $e(m,n)$ *instead of* $a_{mnkl}$.
(ii) $A\in(B(\mathcal{C}_{bp}):\mathcal{CS}_{\vartheta})$ *if and only if* (3.1)-(3.3), (3.4) *and* (3.5) *hold with* $e(m,n)$ *instead of* $a_{mnkl}$.
(iii) $A\in(B(\mathcal{C}_{r}):\mathcal{CS}_{\vartheta})$ *if and only if* (3.1)-(3.3), (3.8) *and* (3.9) *hold with* $e(m,n)$ *instead of* $a_{mnkl}$.
(iv) $A\in(B(\mathcal{L}_{q}):\mathcal{CS}_{bp})$ *if and only if* (3.2) *and* (3.11) *hold for* $1< q<\infty$ *with* $e(m,n)$ *instead of* $a_{mnkl}$.
(v) $A\in(B(\mathcal{L}_{q}):\mathcal{CS}_{bp})$ *if and only if* (3.2) *and* (3.10) *hold for* $0< q\leq1$ *with* $e(m,n)$ *instead of* $a_{mnkl}$.
(vi) $A\in(B(\mathcal{L}_{q}):\mathcal{BS})$ *if and only if* (3.10) *holds for* $0< q<1$ *with* $e(m,n)$ *instead of* $a_{mnkl}$.
(vii) $A\in(B(\mathcal{L}_{q}):\mathcal{BS})$ *if and only if* (3.11) *holds for* $1< q<\infty$ *with* $e(m,n)$ *instead of* $a_{mnkl}$.
(viii) $A\in(B(\mathcal{M}_{u}):\mathcal{CS}_{bp})$ *if and only if* (3.1), (3.3), (3.12), (3.13),(3.14) *and* (3.15) *hold with* $e(m,n)$ *instead of* $a_{mnkl}$.
(ix) $A\in(B(\mathcal{M}_{u}):\mathcal{CS}_{p})$ *if and only if* (3.2), (3.6) *and* (3.7) *hold with* $e(m,n)$ *instead of* $a_{mnkl}$.
(x) $A\in(B(\mathcal{C}_{bp}):\mathcal{BS})$ *if and only if* (3.1) *holds with* $e(m,n)$ *instead of* $a_{mnkl}$.

We may also give the following results derived from Theorems (4.1), (4.2) and (4.3) of Altay and Başar [17] by using relation (4.6).

### Corollary 4.8

*Suppose that the elements of the four*-*dimensional matrices* $A=(a_{mnkl})$ *and* $G=(g_{mnkl})$ *are connected with relation* (4.6). *Then* $A=(a_{mnkl})\in(\mathcal{CS}_{bp}:B(\mathcal{C}_{p}))$ *if and only if conditions* (3.1) *and* (3.2) *hold with* $\Delta_{11}^{kl}g_{mnkl}$ *instead of* $a_{mnkl}$ *and the following conditions hold*:

4.9 $$\begin{aligned}& \lim_{l\to\infty}\Delta_{10}^{kl}g_{mnkl}=0 \quad \textit {for every fixed }k\in\mathbb{N}\textit{ for all }m,n\in\mathbb {N}, \end{aligned}$$

4.10 $$\begin{aligned}& \lim_{k\to\infty}\Delta_{01}^{kl}g_{mnkl}=0 \quad \textit {for every fixed }l\in\mathbb{N}\textit{ for all }m,n\in\mathbb {N}, \end{aligned}$$

4.11 $$\begin{aligned}& \exists g_{kl}\in\mathbb{C}\ni bp\mbox{-} \lim _{m,n\to \infty}\sum_{l} \bigl\vert \Delta_{10}^{kl}g_{mnkl} \bigr\vert =\sum _{k}|g_{kl}|. \end{aligned}$$

### Corollary 4.9

*Suppose that the elements of the four*-*dimensional matrices* $A=(a_{mnkl})$ *and* $G=(g_{mnkl})$ *are connected with relation* (4.6). *Then* $A=(a_{mnkl})\in(\mathcal{CS}_{r}:B(\mathcal{C}_{p}))$ *if and only if condition* (3.1) *holds with* $\Delta _{11}^{kl}g_{mnkl}$ *instead of* $a_{mnkl}$ *and the following conditions hold*:

4.12 $$\begin{aligned}& (g_{mnk0})_{k\in\mathbb{N}},(g_{mn0l})_{l\in\mathbb {N}} \in bv\quad \textit{for all }m,n\in\mathbb{N}, \end{aligned}$$

4.13 $$\begin{aligned}& \exists L\in\mathbb{N}\ni\Delta _{11}^{kl}g_{mnkl}=0 \quad \textit{for all }k\in\mathbb{N}\textit{ whenever }m,n,l>L, \end{aligned}$$

4.14 $$\begin{aligned}& \exists K\in\mathbb{N}\ni\Delta _{11}^{kl}g_{mnkl}=0 \quad \textit{for all }l\in\mathbb{N}\textit{ whenever }m,n,k>K, \end{aligned}$$

4.15 $$\begin{aligned}& \exists g_{kl}\in\mathbb{C}\ni p\mbox{-} \lim _{m,n\to \infty}\sum_{l} \bigl\vert \Delta_{10}^{kl}g_{mnkl} \bigr\vert =\sum _{k}|g_{kl}|. \end{aligned}$$

### Corollary 4.10

*Suppose that the elements of the four*-*dimensional matrices* $A=(a_{mnkl})$ *and* $G=(g_{mnkl})$ *are connected with relation* (4.6). *Then* $A=(a_{mnkl})\in(\mathcal{CS}_{r}:B(\mathcal{C}_{r}))$ *if and only if condition* (3.1) *holds with* $\Delta _{11}^{kl}g_{mnkl}$ *instead of* $a_{mnkl}$ *and* (4.12) *holds*, *and the following conditions also hold*:

4.16 $$\begin{aligned}& r\mbox{-} \lim_{m,n\to\infty}\Delta _{11}^{kl}g_{mnkl}=g_{kl}\quad \textit{for all }l_{0}\in\mathbb{N}, \end{aligned}$$

4.17 $$\begin{aligned}& r\mbox{-} \lim_{m,n\to\infty}\sum _{k}\Delta _{11}^{kl}g_{mnkl}=u_{l_{0}} \quad \textit{for all }l_{0}\in\mathbb{N}, \end{aligned}$$

4.18 $$\begin{aligned}& r\mbox{-} \lim_{m,n\to\infty}\sum _{l}\Delta _{11}^{kl}g_{mnkl}=u_{k_{0}} \quad \textit{for all }k_{0}\in\mathbb{N}, \end{aligned}$$

4.19 $$\begin{aligned}& r\mbox{-} \lim_{m,n\to\infty}\sum _{k,l}\Delta_{11}^{kl}g_{mnkl}=u. \end{aligned}$$

### Theorem 4.11

$A=(a_{mnkl})\in(B(\mathcal{C}_{p}):\mathcal{C}_{p};p)$ *if and only if*

4.20 $$\begin{aligned}& p\mbox{-} \lim_{m,n\to\infty}e_{mnkl}=0 \quad \textit{for all }k,l\in\mathbb{N}, \end{aligned}$$

4.21 $$\begin{aligned}& p\mbox{-} \lim_{m,n\to\infty}\sum _{k,l}e_{mnkl}=1, \end{aligned}$$

4.22 $$\begin{aligned}& p\mbox{-} \lim_{m,n\to\infty}\sum _{k}|e_{mnkl}|=0\quad \textit{for all }l\in \mathbb{N}, \end{aligned}$$

4.23 $$\begin{aligned}& p\mbox{-} \lim_{m,n\to\infty}\sum _{l}|e_{mnkl}|=0\quad \textit{for all }k\in \mathbb{N}, \end{aligned}$$

4.24 $$\begin{aligned}& \exists v\in\mathbb{C}\ni p\mbox{-} \lim _{m,n\to\infty }\sum_{k,l}|e_{mnkl}|=v \quad \textit{for all }l\in\mathbb{N}, \end{aligned}$$

4.25 $$\begin{aligned}& \sup_{K\in\mathbb{N}}\sum_{k,l>K}|e_{mnkl}|< \infty. \end{aligned}$$

### Corollary 4.12

*Let* $A=(a_{mnkl})$ *be a four*-*dimensional infinite matrix*. *Then the following statements hold*.

(i) $A\in(\mathcal{C}_{p}:B(\mathcal{C}_{p});p)$ *if and only if* (4.20)-(4.25) *hold with* $f_{mnkl}$ *instead of* $a_{mnkl}$.
(ii) $A\in(B(\mathcal{C}_{p}):B(\mathcal{C}_{p});p)$ *if and only if* (4.20)-(4.25) *hold with* $h_{mnkl}$ *instead of* $a_{mnkl}$.
(iii) $A\in(B(\mathcal{C}_{p}):\mathcal{CS}_{p};p)$ *if and only if* (4.20)-(4.25) *hold with* $e(m,n)$ *instead of* $a_{mnkl}$.
(iv) $A\in(\mathcal{CS}_{p}:B(\mathcal{C}_{p});p)$ *if and only if* (4.20)-(4.25) *hold with* $\Delta _{11}^{kl}g_{mnkl}$ *instead of* $a_{mnkl}$.

## Conclusion

Zeltser [18], in her PhD thesis, studied both the theory of topological double sequence spaces and the summability theory of double sequences.

Altay and Başar [17] have recently studied the double series spaces $\mathcal{BS}$, $\mathcal{BS}(t)$, $\mathcal {CS}_{\vartheta}$ and $\mathcal{BV}$ whose sequences of partial sums are in the spaces $\mathcal{M}_{u}$, $\mathcal{M}_{u}(t)$, $\mathcal {C}_{\vartheta}$ and $\mathcal{L}_{u}$, respectively, where $\vartheta \in\{p,bp,r\}$. They studied some topological properties of those spaces and computed the *α*-duals of the spaces $\mathcal{BS}$, $\mathcal{CS}_{bp}$ and $\mathcal{BV}$ and the $\beta(\vartheta )$-duals of the spaces $\mathcal{CS}_{bp}$ and $\mathcal{CS}_{r}$ of double series. Furthermore, they gave the conditions which characterize the classes of four-dimensional matrix transformations defined on the spaces $\mathcal{CS}_{bp}$, $\mathcal{CS}_{p}$ and $\mathcal{CS}_{r}$.

Başar [15], Chapter 7, p.277, studied the fundamental results on double sequences and related topics. Başar and Sever [4] deeply studied the Banach space $\mathcal{L}_{q}$ of absolutely *q*-summable double sequences and examined the topological properties. Moreover, they determined the *α*-, $\beta(\vartheta)$- and *γ*-duals of $\mathcal{L}_{q}$, where $1\leq q<\infty$ and $\vartheta\in\{p,bp,r\}$.

The concept of matrix domain was examined by several researchers on some single sequence spaces by using some special matrices. Recently some significant studies have been done by several mathematicians for double sequence spaces and four-dimensional matrices (see [19–22]). In this work, I have studied the domain of four-dimensional generalized difference matrix $B(r,s,t,u)$ on some double sequence spaces and examined some topological properties. Furthermore, I determined the *α*-, $\beta(\vartheta)$- and *γ*-duals of some new double sequence spaces and characterized some classes of four-dimensional matrix transformations related to the new double sequence spaces. As a natural continuation of Yeşilkayagil and Başar [23], one can obtain certain new topological properties concerning the space $B(\mathcal{C}_{f})$ of all almost *B* summable double sequences.
